# An Integrative Approach to the Flavonoid Profile in Some Plants’ Parts of the *Annona* Genus

**DOI:** 10.3390/plants11212855

**Published:** 2022-10-26

**Authors:** Ana Luiza Coeli Cruz Ramos, Ana Carolina do Carmo Mazzinghy, Vinícius Tadeu da Veiga Correia, Bruna Vieira Nunes, Lucas Victor Ribeiro, Viviane Dias Medeiros Silva, Reginaldo Ferreira Weichert, Ana Cardoso Clemente Filha Ferreira de Paula, Isabel Maria Nunes de Sousa, Ricardo Manuel de Seixas Boavida Ferreira, Paula Batista-Santos, Raquel Linhares Bello de Araújo, Júlio Onésio Ferreira Melo

**Affiliations:** 1 Departmento de Alimentos, Faculdade de Farmácia, Campus Belo Horizonte, Universidade Federal de Minas Gerais, Belo Horizonte 31270-901, MG, Brazil; 2 Departamento de Ciências Exatas e Biológicas, Campus Sete Lagoas, Universidade Federal de São João del-Rei, Sete Lagoas 36307-352, MG, Brazil; 3 Departamento de Ciências Agrárias, Instituto Federal de Educação, Ciência e Tecnologia de Minas Gerais, Campus Bambuí, Bambui 38900-000, MG, Brazil; 4 LEAF-Instituto Superior de Agronomia, Universidade de Lisboa, 1649-004 Lisboa, Portugal

**Keywords:** flavonoids, *Annona crassiflora* Mart., *Annona muricata*, *Annona cherimolia* Mill × *Annona squamosa* L., *Annona squamosa*, *Annona coriacea*

## Abstract

The Annonaceae family is widely distributed in subtropical and tropical regions. Several species of this family are known for their pharmacological and beneficial properties to human health, mainly attributed to flavonoids. The objective of this work was to carry out an integrative review in order to identify the main flavonoids found in some plant parts belonging to the *Annona* genus: araticum tree (*Annona crassiflora* Mart.), graviola tree (*Annona muricata*), atemoya tree (*Annona cherimolia* Mill × *Annona squamosa* L.), pinha tree (*Annona squamosa*), bananinha tree (*Annona leptopetala*), and marolinho tree (*Annona coriacea*). Only articles published between the years 2016 to 2021 that answered the guiding question were considered, in order to obtain recent data. Then, search strategies were designated for each database used: Science Direct, CAPES Periodicals, and Scielo. Most of the studies retrieved from the databases are related to fruits. The results showed that the number of flavonoids identified varies according to the analytical methodology used to identify and quantify the compounds. Quercetin was the most commonly found compound in all fruits of the *Annona* genus studied, and epicatechin, rutin, and kaempferol were also found to a lesser extent. The presence of these compounds in *Annona* makes the fruit promising, with potential applicability in the pharmaceutical and food industry.

## 1. Introduction

The Annonaceae family is widely distributed in subtropical and tropical regions. This family comprises a great diversity [1], with 180 genera and more than 3000 species. It stands out as the most prominent family in the Magnoliales order. The presence of many primitive morphological features and the ability to survive mass extinctions have characterized Annonaceae species as “living fossils” [2].

The botanical characteristics of the Annonaceae family may differ from species to species, depending on the origin, climate, and topography. This diversity can range from trees to shrubs to perennial vines, with elongated cylindrical intracellular resin channels and a broad and well-developed septate pith in the stems. The aromatic flowers bloom before they are fully developed. They are axillary, singular or grouped, hermaphroditic, and regular in shape. The fruits are made up of bunches of berries and are widely consumed due to their high nutritional value [2,3].

Annonaceae are very famous due to their wide use in traditional medicine. Several pharmacological studies have been conducted related to the medicinal use of the Annonaceae family, such as its use against pain, arthritis, rheumatism, and neuralgia, and in weight loss [4], anthelmintic and malaria [5], and antiplatelet [6] treatments.

This family also stands out for its wide variety of chemical constituents belonging to various phytochemical groups. Studies report the presence of cyclopeptides [7], flavonoids [8,9], terpenoids [10,11], lignans [12,13], steroids [14], tannins [8], volatile oils, resins [12], many alkaloids [15,16], and acetogenins [17]. Commercially, the most important Annonaceae are the species of the genus *Annona* [18]. Among the species of this genus, we can highlight plants such as pinha, marolo, graviola [19], araticum [20], marolinho [21], and bananinha [22].

**Figure 1:**
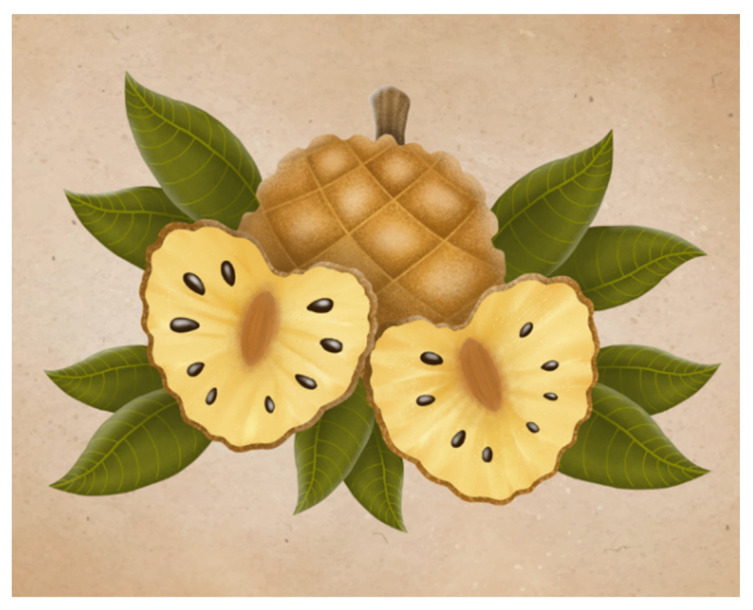
Araticum (*Annona crassiflora* Mart.). Source: Illustration by Ribeiro, L.V., 2022.

Araticunzeiro plants are heterogeneous in terms of stem diameter (10.4 cm), plant height (3.7 m), and production (average of six fruits per plant), with production being the most variable characteristic [23]. The tree produces fruits with acceptable sensory characteristics, as well as significant nutritional and functional potentials [24], and their fruit is the most studied part of this plant. In this vein, the Araticum (*Annona crassiflora* Mart.) (Figure 1) is a fruit-bearing species native to the Brazilian Cerrado biome. When ripe, the araticum fruit has a flattened circular shape, rigid epicarp, and brown coloration [25]. The fruit has an oval or rounded shape, weighing from 0.5 to 4.5 kg, and its seeds are dark brown with a flat oval shape [26]. The harvest of this fruit takes place between September and April [27,28] and provides a great nutritional richness and a wide diversity of phytochemicals, such as carotenoids, folate, flavonoids, ascorbic acid, and vitamins A and E [29,30]. The fruit has reduced total titratable acidity (0.51 g citric acid/100 g) and pH (4.64) and high levels of total soluble solids (13 Brix) and moisture (74.30%), allowing its use in desserts such as sweets, jams, and yogurts. The fruit pulp contains high levels of carbohydrates (18.65%), lipids (3.78%), and energy value (113.65 kcal/100 g), and relatively low amounts of proteins (1.27%) [24].

**Figure 2:**
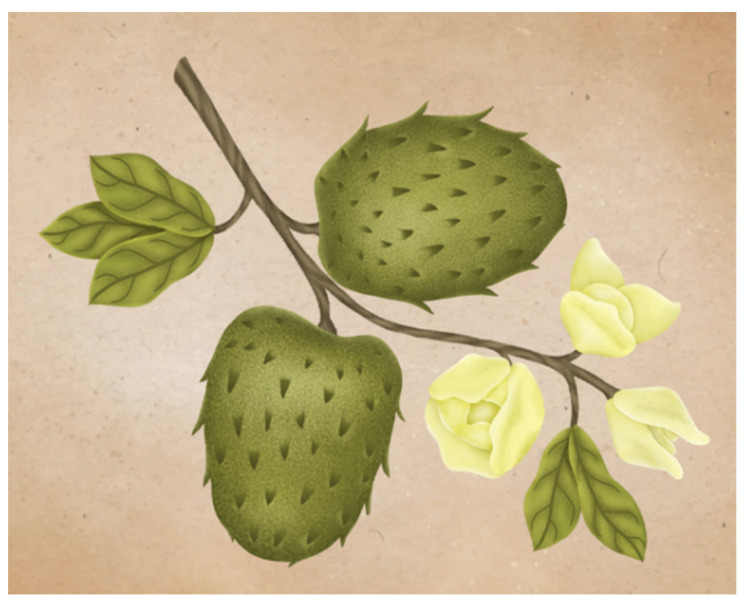
Graviola (*Annona muricata*). Source: Illustration by Ribeiro, L.V., 2022.

The fruit and parts of the tree (leaves, stem, and peel) are the subjects of numerous research works due to their content of potentially bioactive compounds [31]. The biological activities of infusions, decoctions, or extracts of *A. muricata* leaves are related to their content of bioactive compounds, and these leaves are considered a rich source of bioactive compounds with potential uses in the formulation of therapeutic drugs or nutraceutical foods [32]. The fruit of the gravioleira tree, graviola (*Annona muricata*) also known as soursop (Figure 2), is a native fruit of North and South America and is currently distributed in subtropical and tropical regions. Its fruits are large and green in color [33]. The fruiting period of graviola generally occurs throughout the year; however, depending on the altitude, there are more defined seasons [34]. This species has a high concentration of secondary metabolites, such as flavonoids, terpenoids, alkaloids, and some lactones, in addition to minerals such as potassium, calcium, sodium, iron, and magnesium [35]. According to Fuentes (2021), in a bibliographic survey, the nutritional composition of soursop ranges from 55.4 to 81.83 (kcal) for total energy, 80.48–83.2% for moisture content, 0.69–1.10% for protein, 0.20–0.97% for lipid content, 12.50–18.23% for soluble carbohydrates, and 4.83–5.76% for dietary fiber content [36].

**Figure 3:**
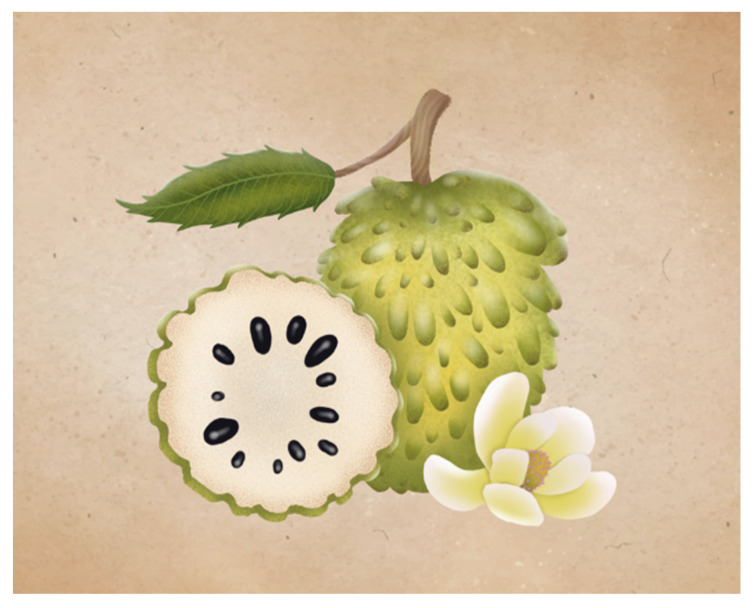
Atemoya (*Annona cherimola* Mill. × *Annona squamosa* L.). Source: Illustration by Ribeiro, L.V., 2022.

The atemoya tree a hybrid plant resulting from the cross between cherimoya (*A. cherimola* Mill.) and pinha (*A. squamosa* L.) [37] maintains the good qualities of both plants. The atemoya fruit (*Annona cherimola* Mill. × *Annona squamosa* L.) (Figure 3) has a small number of seeds, better conservation after harvest, absence of cracks and resistance to weeds, and improved biological control [38]. When pollinated, its shape is heart-shaped, ovate, or conical. The surface of the atemoya is smooth or may present prominence, and the pulp of this fruit has a white color. Regarding its phytochemicals, reports in the literature place the greatest emphasis on the class of acetogenins [39]. This fruit has approximately 65.18% moisture, 0.55% lipids, 1.24% proteins, 2.04% ash, 31.99% carbohydrates, and 1.55% total fiber [38]. Its leaves and stems stand out for their total phenol and flavonoid levels, as well as their antioxidant and antimicrobial activities [40].

**Figure 4:**
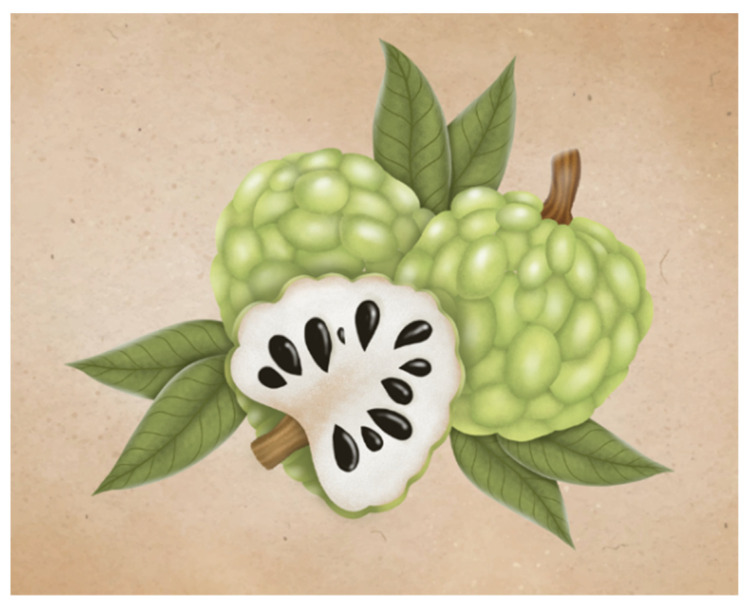
Pinha (*Annona squamosa* L.). Source: Illustration by Ribeiro, L.V., 2022.

The pine tree (*Annona squamosa*) is characterized by its small size, pivoting roots, and elongated leaves. These features also have several ramifications. The height of this plant varies from three to six meters, its flowers are small and can be isolated or in the form of a bunch, and its fruits are flat, ovoid, or heart-shaped [41]. The *Annona squamosa* L. fruit (Figure 4), popularly known as “ata”, “pinha”, or “fruta-do-conde”, is widely used for fresh consumption [42]. The fruit is widely known and consumed according to its medicinal and nutritional properties and its pleasant taste. It stands out for the presence of health-related components such as vitamins (A, B, C, E, and K1), antioxidants, polyunsaturated fatty acids, and the presence of essential minerals. In addition, this plant has a variety of compounds responsible for various activities such as the inhibition of insect attack, antimalarial, cytotoxic, immunosuppressive, and anti-HIV properties, and antiplatelet activity [43]. This fruit has an average caloric content of 95.3 (kcal), 70% moisture, 1.23% proteins, 0.21% lipids, 22.8% soluble carbohydrates, and 1.02% dietary fiber [44].

**Figure 5:**
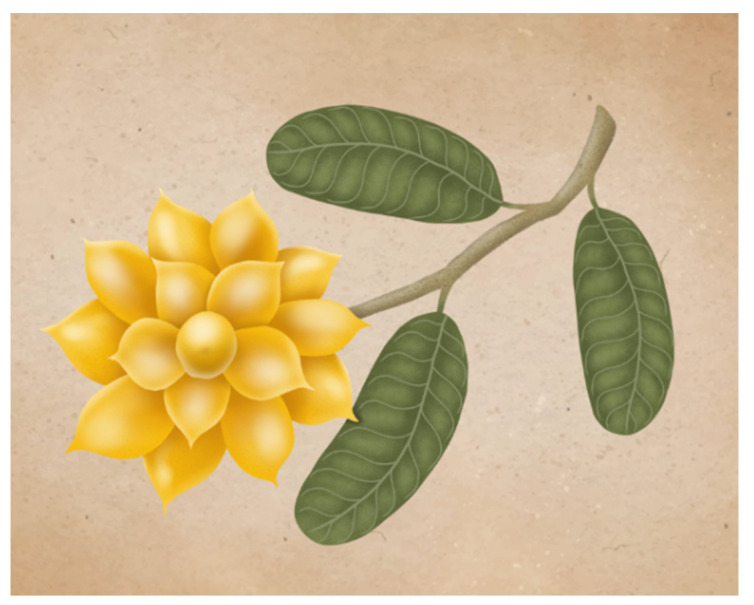
Bananinha (*Annona leptopetala*). Source: Illustration by Ribeiro, L.V., 2022.

*Annona leptopetala* is considered an endemic Brazilian tree. It is a woody, deciduous species with a high representation. This species’ reproductive and vegetative development is associated with the availability of water in nature [45,46]. The fruit (Figure 5), popularly known as “bananinha” [47], is the second most abundant species in the Brazilian Cerrado [46]. This species is unique in the genus, which has red flowers. From 2 to 9 m in height, its tree has elliptical to ovate leaves, with yellow, orange, or red apocarpous fruits, its flowering and fruiting ranges from November to April [47]. Bananinha is indicated in traditional medicine for the treatment of inflammation and tumors [48], and for its larvicidal potential [49], as well as its modulating effect on bacterial resistance to norfloxacin, in addition to a considerable antitumor effect in vivo [50]. The phytochemical study points to the presence of aporphine and tetrahydro-protoberberine alkaloids, in addition to acetogenins and a xanthone [51].

**Figure 6:**
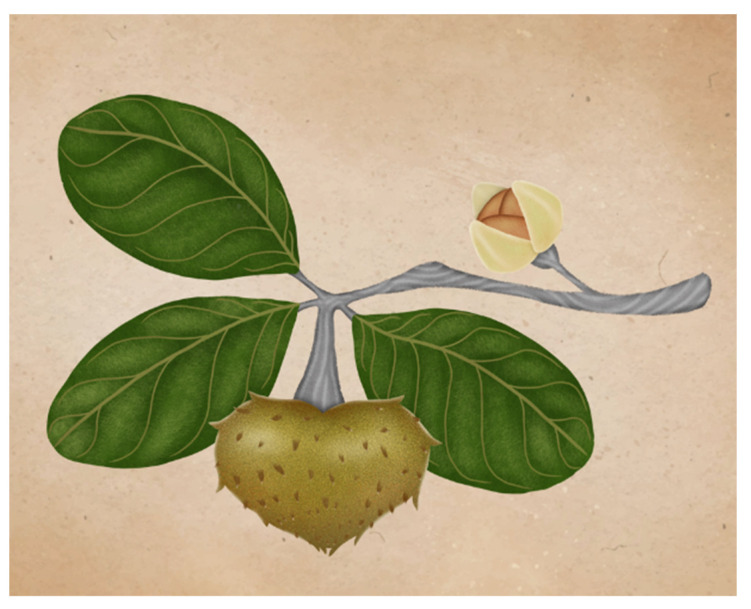
Marolinho (*Annona coriácea*). Source: Illustration by Ribeiro, L.V., 2022.

*Annona coriacea* is considered an evergreen shrub or small tree [21]. Popularly known as “marolinho” and “cabeça-de-negro”, the species *Annona coriacea* occurs in some states of northeastern Brazil in an area of caatinga [52]. The height of this tree species varies from 3 to 18 m, with simple, obovate leaves, glabrous on the ventral surface, the base is often chordate and the margin wavy; the flowers are solitary, terminal, and bushy, with fleshy petals ranging in color from orange to orange-pink [21,53]. The fruit (Figure 6) is globular or elongated, white pulp, watery, soft, smooth rind, or fleshy scales. In Brazil, it is popularly used against chronic diarrhea, malaria, helminths, and leishmaniasis [53]. Many biological activities are described for *A. coriacea*, including insecticidal activity [50,54], antiprotozoal [55], phytotoxic [56], cytoprotective [57], antiproliferative, cytotoxic, and enzymatic inhibitory effects [58]. Phytochemical studies report the presence of polyphenols [57], alkaloids [59], and volatile compounds [60].

Several reports in the literature show that such varieties are correlated with antitumor, antioxidant, and antiparasitic activities, as well as antimicrobial and anti-inflammatory effects [24,60,61]. Anaya-Esparza and collaborators (2020) show in a bibliographical review that *Annona* species are widely used in folk medicine, demonstrating great importance. Their traditional use is associated with treating fever, diarrhea, dysentery, hematuria, urethritis, asthma, and parasitic and hepatic diseases, in addition to exerting antispasmodic, laxative, antiemetic, antisudorific, antitussive, antiflu, and antidepressant effects [62]. Several of these species are well known due to their pharmacological properties and benefits to human health, mainly attributed to the presence of flavonoids, the main bioactive constituent found within them [18].

**Figure 7:**
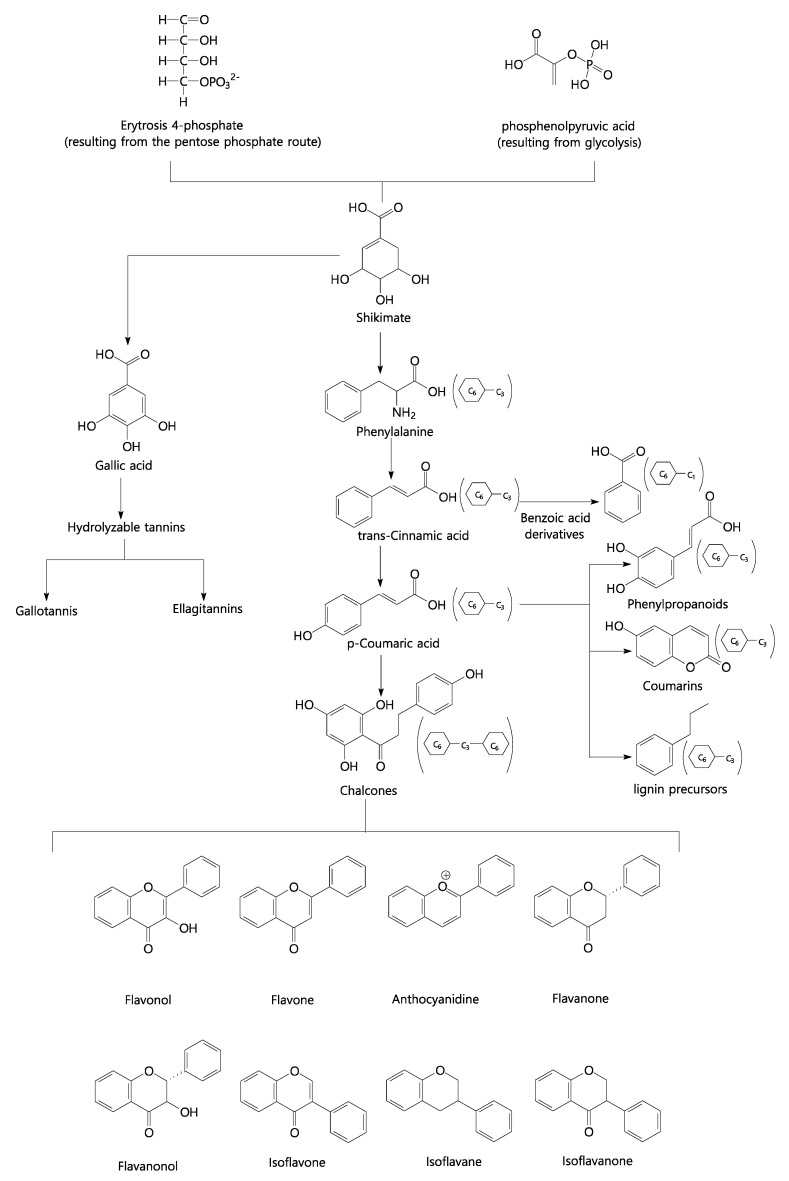
Simplified biosynthetic pathway of phenolic compounds. Source: Authors, 2022.

Phenolic compounds belong to a large compound class that includes both simple and complex structures with at least one aromatic ring. We can highlight several derived classes of phenolic compounds and their basic skeleton: C6 simple phenols, benzoquinones; C6-C1 phenolic acids; C6-C2 acetophenones and phenylacetic acids; C6-C3 phenylpropanoids; C6-C3 coumarins; C6-C4 naphthoquinones; C6-C1-C6 xanthones; C6-C2-C6 stilbenes and anthraquinones; C6-C3-C6 flavonoids and isoflavonoids; (C6-C3)2 lignans; (C6-C3-C6)2 diflavonoids; (C6)n plant melanins; (C6-C3)n lignins; (C6-C1)n atemoya tannins; (C6-C3-C6)n condensed tannins. Figure 7 shows the simplified biosynthetic route of phenolic compounds, highlighting flavonoids.

Flavonoids are part of the most relevant and diversified group of phenolic compounds among natural products [63] and are widely distributed in the plant kingdom [64,65]. They stand out for having great economic attractiveness due to their various properties beneficial to health [66]. In the *Annona* genus, the most common flavonoid classes are quercetin-3-*O*-rhamnoside, luteolin-7-*O*-glucoside, kaempferol-3-*O*-galactoside, and their derivatives [67].

These compounds belong to an important class of secondary metabolites [68], in addition to having the virtue of their wide variety of biological and therapeutic activities, demonstrated both in vitro and in vivo [65]. We can thus highlight the correlation of these compounds with antioxidant, anti-inflammatory, antitumor, and antiviral properties, among others [60].

Therefore, it is essential to know which flavonoids are present in these plants to appreciate these species better. Therefore, the objective of this work was to carry out an integrative review to identify which flavonoids are mostly found in plant parts belonging to the *Annona* genus.

## 2. Results and Discussion

The definition of the guiding question led to the designation of search strategies for each database used, namely Science Direct, Periódicos CAPES, and Scielo. The descriptors were used as a search strategy that approached the popular and scientific names of the six *Annona* plants selected for the present work, associated or not with the term “flavonoid”, according to each database.

It was possible to refine the search for articles related to flavonoids in plants of the *Annona* genus using AND/OR search operators. The pre-selection was based on the relationship of guiding questions and reading both the abstract and the title of the works. The search strategy used for the Science Direct database resulted in 113 studies, while 63 articles were found in Periódicos Capes and 41 studies were selected in the Scielo platform. Of the 217 works retrieved, 8 were removed because they appeared twice in the databases. In total, 209 works were selected for the next stage (Table 1).

**Figure 8:**
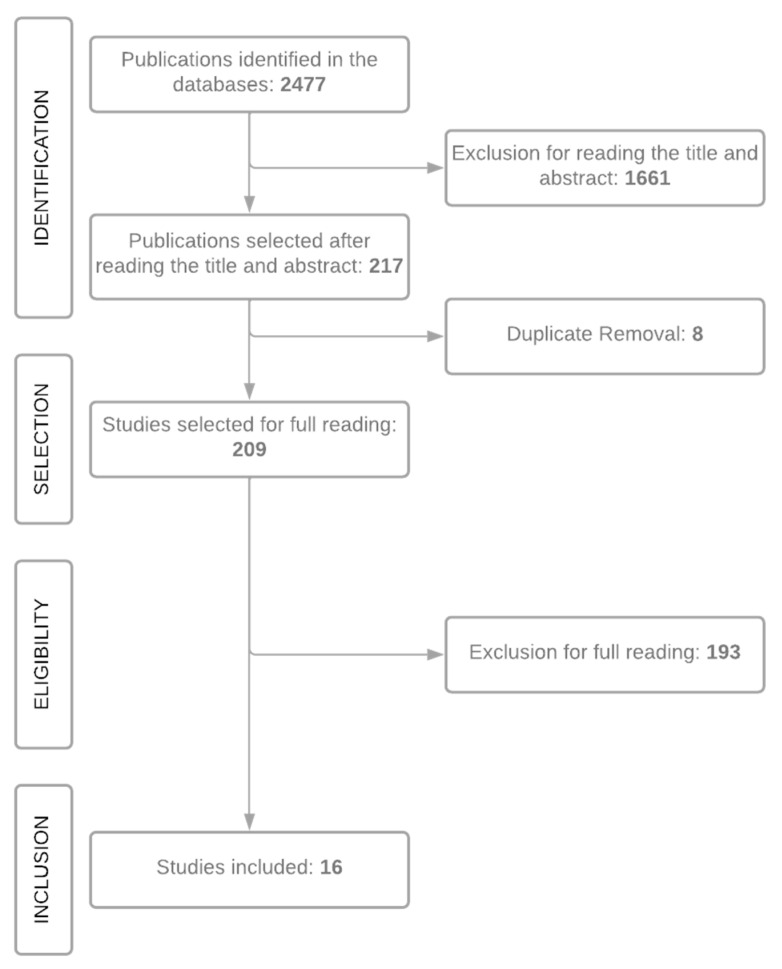
Flowchart of the selection of studies by stages. Source: Authors, 2022.

Subsequently, a complete reading of the 209 pre-selected articles was necessary, followed by the interpretation of the data that answered the guiding question of this review. The selection process was developed as shown in Figure 8.

Table 2 briefly presents what was performed in the 16 selected studies. It can be observed that approximately 43.75% (7) were related to *Annona muricata*, 31.25% (5) to *Annona crassiflora* Mart., 18.75% (3) to *Annona coriacea*, and 6.25% (1) to *Annona cherimolia* Mill × *Annona squamosa* L. It is worth mentioning that one of the studies recovered was related to more than one (graviola and araticum). Studies related to *A. squamosa* and *A. leptopetala* were not included due to the lack of studies returned in the survey that answered the guiding question.

It can be observed that the flavonoids most commonly found in araticum (*A. crassiflora* Mart.) were epicatechin, quercetin, and rutin. Among the flavonoids identified in graviola (*A. muricata*), rutin, quercetin, and kaempferol stand out. Regarding the compounds isolated from marolinho (*A. coriacea*), quercetin was the most abundant (Table 2). The presence of flavonoids gives araticum important biological activities such as antitumor, antioxidant, anti-inflammatory, antidiarrheal, analgesic, antimicrobial, and antiparasitic activities [57].

The main method of flavonoid extraction used by these authors, and those of the other studies retrieved, was HPLC-ESI-MS/MS. However, the amount and type of flavonoid identified varied according to the methodology used. Quercetin and epicatechin were the main compounds found in all studies, but also, to a lesser extent, catechin, rutin, and kaempferol-based structures were found.

Arruda et al. found that in araticum pulp, peel, and seed, the main flavonoids were catechin, epicatechin, and caffeic acid. According to these authors, compounds with antioxidant activity act by deactivating free radicals through two main mechanisms: transfer of a single electron (ability to transfer an electron to reduce any compound, including radicals, metals, and carbonyls) and transfer of hydrogen atoms (ability to quench free radicals by donating hydrogen) [26]. Similarly, the review presented by Arruda and Pastore addressed several studies that identified quercetin and epicatechin in araticum leaves [24], emphasizing its potential use as a plant ingredient in new drugs and cosmetic formulations, while Justino et al. identified these compounds in the fruit peel, mentioning the possibility of blocking carbohydrate digestive enzymes and the formation of glycation products [70]. According to these authors, the presence of these flavonoids in the species, in addition to conferring biological activities, suggests that the identification and isolation of these phytochemicals may be useful for applications in nutraceutical supplements, food additives, and pharmaceuticals.

On the other hand, Prado et al. found a higher flavonoid content in the peel than in the seed in a quantitative investigation of flavonoids in different fruit parts [69]. Accordingly, Arruda et al. reported that most flavonoids were found in the peel of the araticum fruit [71]. However, they were also identified in the pulp and seed of araticum. This more significant presence of flavonoids in the peel can be explained by the role of these compounds in participating in the plant’s defense against UV radiation and the attack of herbivores, fungi, and viruses [70].

Concerning graviola, Souza et al. worked with a hydroalcoholic extract of *A. muricata* leaves due to an interest in the neuroactive properties of its flavonoids in mice [77]. An HPLC analysis of the extract revealed the presence of flavonoids (quercetin, isoquercitrin, rutin, and kaempferol). Quercetin and kaempferol were classified as anxiolytic constituents with sedative effects, which justifies the use of this plant to relieve anxiety, depression, and sleep disorders.

Lee and colleagues, who also evaluated graviola leaves, found that in addition to quercetin and some of its derivatives, there was also the presence of aponarin, epicatechin, and glabridin [31]. These compounds showed a high antioxidant activity, supporting the use of graviola leaves for food applications. Using the LC-MS/MS technique, Kim et al. found the flavonoids kaempferol-3-*O*-rutinoside and quercetin-3-*O*-rutinoside in a graviola leaf extract, in addition to demonstrating the ability of soursop leaves to boost immunity [75]. Similarly, Balderrama-Carmona et al. identified rutin as the predominant compound through analyses performed using the UPLC technique, and reported that graviola leaves can help reduce oxidative stress in human erythrocytes, as well as viral infections [78]. Thus, it is possible to observe that, regardless of the technique used, rutin, kaempferol, quercetin, and their derivatives are prominently present in *A. muricata*.

A study by Nam et al. focused on comparing the antioxidant activity of different extracts and parts (roots, branches, and leaves) of graviola [76]. The 80% methanol extracts showed a content of flavonoids (in particular epicatechin and rutin), almost six times higher, with all parts than the distilled water extracts. Among the 80% methanol extracts, the leaves had the highest flavonoid content. According to these authors, a higher flavonoids content in the leaves may be due to the accumulation of polyphenols such as catechin generated during photosynthesis.

Research by Justino et al. evaluated the antidiabetic potential of graviola leaves and performed an ethanolic extraction followed by a liquid–liquid partition of the ethanolic extract [74]. As a result, the ethyl acetate fractions showed a high content of flavonoids, including procyanidins B2 and C1, (epi)catechin, quercetin, and kaempferol. In this way, these results present new biological activities for the fruit, which are associated with understanding the potential effectiveness of graviola leaf for managing diabetes mellitus and its complications.

In addition to the most consumed parts of the fruits, such as pulp and leaves, large amounts of waste such as seeds are discarded during fruit processing, generating a deficit of information on the exploitation of these by-products [73]. In this way, there is a demand for the greater characterization of these unconsumed parts. Thus, Menezes and collaborators evaluated the profile of the bioactive compounds of graviola seed and found that rutin was the main flavonoid found in the by-product of this fruit [73]. In this way, graviola seeds also become potentially exploitable for the food and/or pharmaceutical sector.

When exploring the nutraceutical potential of atemoya (*A. squamosa* × *A. cherimola*), Mannino and colleagues, in a 2020 study, evaluated the chemical profile of this fruit and observed that among the compounds identified, six were from the flavonoid class [62]. The compounds identified in the greatest abundance were quercetin and kaempferol. This work also suggests the potential use of *A. cherimola* leaves to prepare dietary supplements to promote the physiological redox balance that plays an essential role in preventing several chronic diseases [62].

Rocha et al. carried out data collection through a systematic review to identify the flavonoids present in the species *A. coriacea* [21]. As a result, these authors identified high concentrations of luteolin and quercetin present in the fruit, and those flavonoids are the second class of secondary metabolites with the highest reports for this fruit. The presence of these compounds gives the species significant biological activity, emphasizing the anti-inflammatory, anticancer, insecticidal, and antiulcer activities, the latter being associated with the cytoprotective, anti-secretory, and antioxidant capacity of these bioactive compounds.

**Figure 9:**
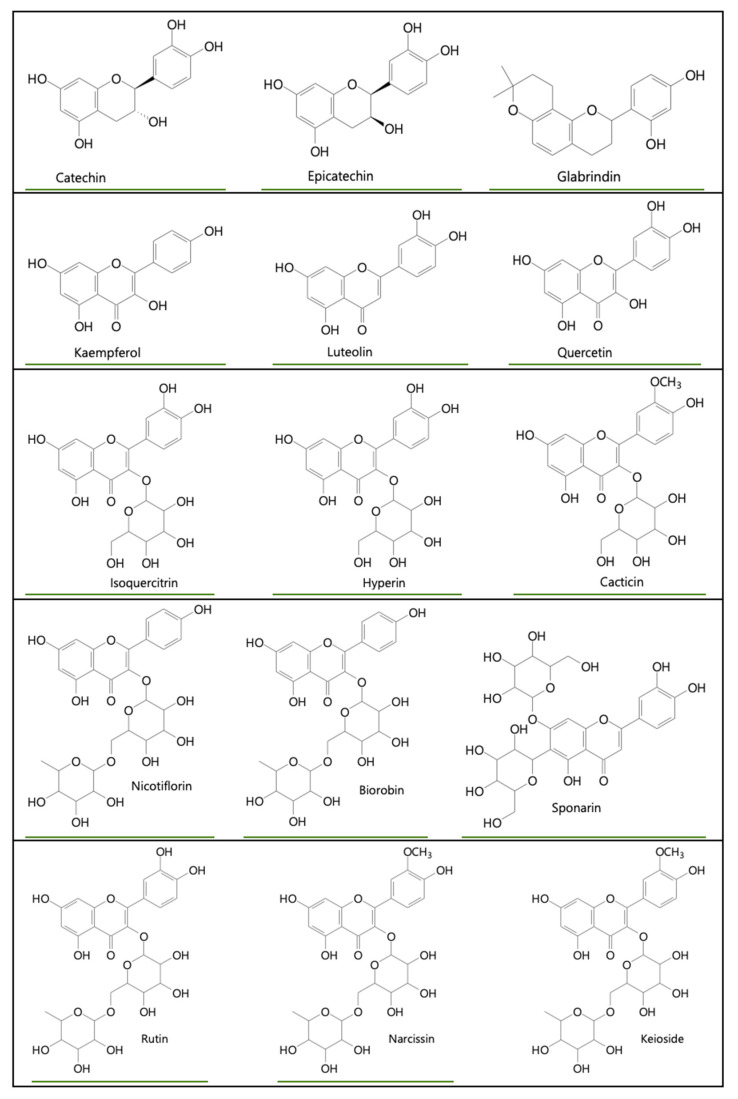
Main flavonoids found in the genus *Annona*. Source: Authors, 2022.

Using traditional UV spectroscopy techniques, Novaes et al. identified in *A. coriacea* leaves the presence of the quercetin, found in a more significant proportion, followed by kaempferol and isorhamnetin [72]. In 2019, Novaes and collaborators identified the presence of eleven flavonoids in the leaves of marolinho (*A. coriacea*), emphasizing quercetin-3-*O*-gentiobioside and quercetin-3-*O*-robinobioside in a similar way to the previous study, and also for rutin [62]. According to these authors, such compounds are correlated with the nutraceutical properties of marolinho and their wide applicability for the preparation of functional foods and the formulation of dietary products. Figure 9 shows the main flavonoids found in plants of the genus *Annona*.

In general, the Annonaceae family, especially the fruits discussed in the present study, have a great prominence of compounds such as quercetin, rutin, and kaempferol, since these compounds were present in 93.75% of the included studies. Concomitantly, it can be inferred that even carrying out the research of these components through different methodologies and technologies, flavonoids are always present in species of the genus *Annona*. Therefore, these studies emphasize the importance of these species, since they can bring several health benefits and have a wide application in the food and pharmaceutical industry, especially *Annona crassiflora* Mart. (araticum), *Annona muricata* (graviola), *Annona cherimolia* Mill × *Annona squamosa* L. (atemoya), and *Annona coriacea* (marolinho).

## 3. Materials and Methods

Articles published between the years 2016 to 2021 were considered, to obtain recent data, and the searches were performed using three databases, namely: Science Direct, Periódicos CAPES, and Scielo. Initially, six plants belonging to the *Annona* genus were established: araticum (*Annona crassiflora* Mart.), graviola (*Annona muricata*), atemoya (*Annona cherimolia* Mill × *Annona squamosa* L.), pinha (*Annona squamosa*), bananinha (*Annona leptopetala*), and marolinho (*Annona coriacea*), defined in light of the previous research about them.

The study was carried out in five stages according to the methodology described by Ramos et al. (2022) [80]. The first step consisted of elaborating a guiding question, defined as: “Which flavonoids are mostly found in plant parts belonging to the *Annona* genus?”. In the second stage, searches were carried out, based on predetermined strategies (Table 3), for articles in the three databases.

Inclusion criteria were studies published between 2016 and 2021 that identified flavonoids present in plant parts of the *Annona* genus mentioned above. There was no restriction regarding the language of the articles, and only scientific articles were used. Exclusion criteria were applied to those articles that did not answer the guiding question initially asked and those that were not published within the stipulated period. Other types of works returned by the databases were also excluded, such as theses, dissertations, and book chapters. The exclusion criteria were also applied for those articles that did not have free access.

In the third stage, the pre-selection of scientific works was carried out. Both the title and the abstract of each article were read according to the established criteria of inclusion and exclusion. Thus, the complete reading of the pre-selected manuscripts in the previous step was carried out in the next step. As a fifth and final step, the results were interpreted. Only those works which answered the initial guiding question and could aid in developing its answer remained.

## 4. Conclusions

Among the six plants surveyed, the majority that answered the guiding question were related to the fruit, and the plant with the highest number of works was graviola (*Annona muricata*). The selected works also identified flavonoids in araticum (*Annona crassiflora* Mart), marolo (*Annona coriacea*), and atemoya (*Annona cherimolia* Mill × *Annona squamosa* L.). According to the studies selected in this review, it is noticeable that quercetin is the most recurrent flavonoid in all plants of the *Annona* genus addressed in this study. In addition to quercetin, epicatechin, rutin, and kaempferol were reported in most of the studies addressed. The presence of these compounds makes these fruits promising and with potential applicability in the pharmaceutical and food industry due to the different properties they present.

## Figures and Tables

**Table 1:** Works retrieved by searching the databases.

Database	Number of Works	Duplicates	Total
Periódicos Capes	63		
Scielo	41	8	209
Science Direct	113		

Source: Authors, 2022.

**Table 2:** Summary of selected works.

Sample	What Was Done	Flavonoids Found	Reference
Peel extracts of araticum and seeds	This analysis was performed using an HPLC system coupled to a triple quadrupole mass spectrometer equipped with electrospray ionization (ESI).	Epicatechin and quercetin	[69]
Different botanical parts of araticum (fruit, leaves, stem, and root)	A review of the scientific literature was carried out on the main phytochemicals of different botanical parts of *Annona crassiflora* Mart. (fruits, leaves, stem, and root).	Catechin, epicatechin, rutin, quercetin	[24]
Araticum pulp, peel, and seed	The phenolics present in the araticum pulp, peel, and seed were characterized and quantified using HPLC-ESI-MS/MS.	Catechin, epicatechin, and caffeic acid	[26]
Peel of araticum	An HPLC-ESI-MS/MS analysis was performed to identify the main bioactive compounds of *A. crassiflora* fruit peel from ethanol extract fractions with antioxidant capacity and *α*-amylase, *α*-glucosidase, and glycosidase inhibitory activities.	Epicatechin, quercetin glucosides, and kaempferol	[70]
Different partos of marolinho	A search was carried out for scientific articles from electronic databases (Science Direct, PubMed, Lilacs, Scopus, Google Scholar, Scielo, and Web of Science), identifying studies published before November 2020.	Luteolin and quercetin	[21]
Peel of araticum	The effects of rated ultrasonic power (160–640 W) and process time (0.5–5.0 min) on the recovery of phenolic compounds and antioxidant activity of araticum peel were investigated.	Epicatechin, rutin, and catechin	[71]
Leaves of marolinho	The compounds were analyzed using traditional UV spectroscopy techniques and subjected to acid hydrolysis. The identity of eleven isolated compounds was established by ^1^H and 13 RNH spectra and compared with literature data.	Quercetin-3-*O*-*β*-(6″-*O*-*β*-glucosyl)-gloside (quercetin-3-*O*-gentiobioside); quercetin-3-*O*-*β*-(6″-*O*-*α*-rhamnosyl)-galactoside (quercetin-3-*O*-robinobioside); quercetin-3-*O*-*β*-(6″-*O*-*α*-rhamnosyl)-glucoside (rutin), quercetin-3-*O*-*β*-galactoside (hyperin), quercetin-3-*O*-*β*-glucoside (isoquercitrin); kaempferol-3-*O*-*β*-(6″-*O*-*α*-rhamnosyl)-galactoside (biorobin); kaempferol-3-*O*-*β*-(6″-*O*-*α*-rnoshamyl)-glucoside (nicotiflorin); isorhamnetin-3-*O*-*β*-(6″-*O*-*α*-rhamnosyl)-galactoside (chioside); isorhamnetin-3-*O*-*β*-(6″-*O*-*α*-rhamnosyl)-glucoside (narcissin); isorhamnetin-3-*O*-*β*-galactoside (cacticin); isorhamnetin-3-*O*-*β*-glucoside	[72]
Graviola and marolo seeds	Chemical composition was evaluated: approximate analysis, mineral profile, pectic substances, carbohydrates, fatty acid profile, and bioactive compounds of two main exotic fruit residues (seeds), marolo and graviola.	Rutin	[73]
Leaves of graviola	Ultrasound mechanical applicator-assisted extraction (UMSAE) of extraction yield, total alkaloid content (TAC), and antioxidant activity (DPPH) tests were performed.	Saponarin, epicatechin, rutin, kaempferol, glabridin, quercetin, and quercetin-3*β*-*D*-glucoside	[31]
Leaves of graviola	The identification of the main bioactive compounds of *A. muricata* leaves was performed by HPLC-ESI MS/MS.	Epicatechin, quercetin, quercetin-hexolate, and kaempferol	[74]
Leaves extract of graviola	The immune-boosting activity of graviola leaf extracts on RAW 264.7 macrophage cells was examined. The identification of compounds was through LC-MS/MS.	Kaempferol-3-*O*-rutinoside and quercetin-3-*O*-rutinoside	[75]
Different parts of graviola	The active components were evaluated using high-performance liquid chromatography (HPLC) to identify potential development as new functional products.	Rutin and epicatechin	[76]
Leaves of graviola	Flavonoids and phenolic compounds were identified and quantified by the high-performance liquid chromatography (HPLC) method.	Quercetin, isoquercitrin, quercitrin, rutin, and kaempferol	[77]
Leaves extract of graviola	The aqueous extract (AE) and acidified ethanolic extract (AEE) of graviola leaf were characterized by ultra-performance liquid chromatography (UPLC).	Rutin	[78]
Leaves of atemoya	To identify and quantify phenolic compounds in the samples, HPLC-DAD ESI-MS/MS was used.	Quercetin-3-*O*-rutinoside-7-*O* glycoside; quercetin-3-*O*-rutinoside-7-*O*-pentoside; quercetin-3-*O*-rutinoside; kaempferol 3-galactoside-7-rhamnoside; quercetin-3-*O*-glucoside; kaempferol-3-*O*-glucoside; luteolin-3-galactoside-7-rhamnos; luteolin-3-glucoside-7-rhamnose; apigenin-8-*C*-glucoside; catechin and epicatechin	[62]
Leaves of marolinho	The isolated compounds and their commercial aglycones were evaluated for DPPH, ABTS+- radical scavenging, ferric reducing antioxidant power (FRAP), and oxygen reducing antioxidant capacity (ORAC).	Quercetin-3-*O*-gentiobioside; quercetin-3-*O*-robinobioside; rutin; hyperin; isoquercitrin; biorobin; nicotiflorin; keioside; narcissin, cacticin, and isorhamnetin-3-*O*-glucoside	[79]

Source: Authors, 2022.

**Table 3:** Search strategies are used for each database.

Database	Search Strategy	n^o^ of Works
Science Direct	flavonoid AND Araticum OR *Annona crassiflora* flavonoid AND Graviola OR *Annona muricata* flavonoid AND Atemoya OR *A. squamosa* × *A. cherimola* flavonoid AND Pinha OR *Annona squamosa* flavonoid AND Bananinha OR *Annona leptopetala* flavonoid AND *Annona coriacea* OR Marolinho	1177
Periódicos CAPES	Araticum OR *Annona crassiflora* AND flavonoid Graviola OR *Annona muricata* AND flavonoid Atemoya OR *A. squamosa* × *A. cherimola* AND flavonoid Pinha OR (*Annona squamosa*) AND flavonoid Bananinha OR *Annona leptopetala* AND flavonoid *Annona coriacea* OR Marolinho AND flavonoid	1198
Scielo	(Araticum) OR (*Annona crassiflora*) (Graviola) OR (*Annona muricata*) (Atemoya) OR (*A. Squamosa* × *A. Cherimola*) (Pinha) OR (*Annona squamosa*) (Bananinha) OR (*Annona leptopetala*) (Marolinho) OR (*Annona coriacea*)	102

Source: Authors, 2022.

## Data Availability

Not applicable.
